# RNA-binding protein YBX3 promotes PPARγ-SLC3A2 mediated BCAA metabolism fueling brown adipogenesis and thermogenesis

**DOI:** 10.1016/j.molmet.2024.102053

**Published:** 2024-10-29

**Authors:** Lin-Yun Chen, Li-Wen Wang, Jie Wen, Jing-Dong Cao, Rui Zhou, Jin-Lin Yang, Ye Xiao, Tian Su, Yan Huang, Qi Guo, Hai-Yan Zhou, Xiang-Hang Luo, Xu Feng

**Affiliations:** 1 Department of Endocrinology, Endocrinology Research Center, Xiangya Hospital of Central South University, Changsha, Hunan, 410008, China; 2 National Clinical Research Center for Geriatric Disorders, Xiangya Hospital, Changsha, Hunan, 410008, China

**Keywords:** YBX3, Brown adipose tissue, Thermogenesis, Branched-chain amino acid, Obesity

## Abstract

**Objective:**

Activating brown adipose tissue (BAT) thermogenesis is a promising approach to combat obesity and metabolic disorders. The post-transcriptional regulation of BAT thermogenesis mediated by RNA-binding proteins (RBPs) is still not fully understood. This study explores the physiological role of novel RBPs in BAT differentiation and thermogenesis.

**Methods:**

We used multiple public datasets to screen out novel RBPs responsible for BAT differentiation and thermogenesis. In vitro loss- and gain-of-function experiments were performed in both C3H10T1/2 preadipocytes and mature brown adipocytes to determine the role of Y-box binding protein 3 (YBX3) in brown adipocyte differentiation and thermogenesis. Adeno-associated virus (AAV)-mediated BAT-specific knockdown or overexpression of *Ybx3* was applied to investigate the function of YBX3 *in vivo*.

**Results:**

YBX3 is a brown adipocyte-enriched RBP induced by cold stimulation and β-adrenergic signaling. Both *in vitro* loss- and gain-of-function experiments demonstrate that YBX3 is essential for brown adipocyte differentiation and thermogenesis. BAT-specific loss of *Ybx3* dampens thermogenesis and exacerbates diet-induced obesity in mice, while overexpression of *Ybx3* promotes thermogenesis and confers protection against diet-induced metabolic dysfunction. Transcriptome analysis and mitochondrial stress test indicate that *Ybx3* deficiency compromises the mitochondrial oxidative phosphorylation, leading to thermogenic failure. Mechanistically, YBX3 stabilizes the mRNA of *Slc3a2* and *Pparg*, which facilitates branched-chain amino acid (BCAA) influx and catabolism and fuels brown adipocyte differentiation and thermogenesis.

**Conclusions:**

YBX3 facilitates BAT fueling BCAA to boost thermogenesis and energy expenditure, which protects against obesity and metabolic dysfunction. Thus, YBX3 could be a promising therapeutic target for obesity.

## Highlights

• YBX3 is a BAT-enriched RBP in response to ambient temperature and β3-adrenergic/cAMP signaling.
• YBX3 is essential for brown adipocyte differentiation and thermogenesis.
• YBX3 facilitates PPARγ-SLC3A2-mediated BCAA metabolism to fuel brown adipogenesis and thermogenesis.
• YBX3 confers protection against diet-induced obesity and metabolic dysfunction.

## Graphical abstract

**Figure:**
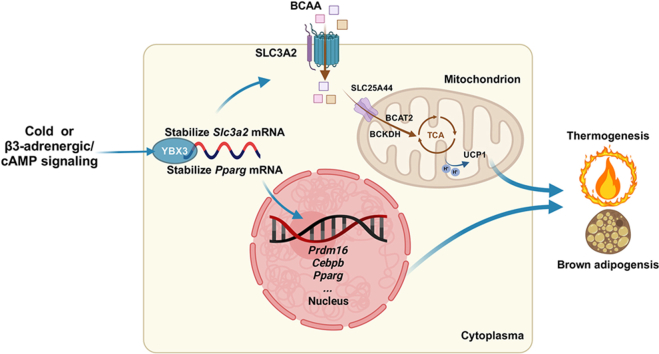

## 1 Introduction

The modern lifestyle contributes to the global prevalence of obesity and related metabolic disorders. However, current therapeutic options remain limited, precipitating the need for novel therapeutic strategies [1]. Obesity is characterized by excessive fat accumulation, which is primarily caused by energy imbalance. Unlike white adipose tissue (WAT), which is majorly specialized for energy storage, brown adipose tissue (BAT) dissipates energy and produces heat through uncoupling protein 1 (UCP1) mediated mitochondrial uncoupling of oxidative phosphorylation [2]. The activation of BAT thermogenesis has been proven efficient in combating obesity and metabolic disorders [[3], [4], [5]] in rodents. Thus, it’s essential to identify the key regulators in brown adipocyte differentiation and thermogenesis.

While brown adipocyte differentiation and thermogenesis have been proven regulated by numerous transcriptional factors at the transcriptional level [[6], [7], [8]], it remains unclear how these processes are post-transcriptionally manipulated by factors including RNA-binding proteins (RBPs). The Y-box binding protein (YBX), including YBX1, YBX2, and YBX3, are canonical RBPs with evolutionarily conserved cold shock domain (CSD) and post-transcriptionally control mRNA translation and mRNA stability [9]. The functions of YBX1 and YBX2 within different adipose depots have been extensively explored. Our previous findings and other groups' studies have demonstrated that YBX1 could enhance mRNA stability across various adipose depots, promoting adipogenesis, thermogenesis, and sympathetic innervation [4,10,11]. Likewise, YBX2 has been reported to stabilize the mRNA of *Ppargc1a* or *Hk2*, facilitating thermogenesis or glycolysis in brown adipocytes [12,13]. Although previous studies indicated that proteins in the YBX family may have overlapping functions [14,15], little is known about the role of YBX3 in adipose biology.

In this study, we integrated multiple public datasets to demonstrate an unannotated role of cold-induced YBX3 in brown adipocyte differentiation and thermogenesis following loss- and gain-of-function experiments. Mechanistically, YBX3 stabilizes the mRNA of *Slc3a2* and *Pparg*, facilitating BCAA influx and catabolism to fuel brown adipocyte differentiation and thermogenesis. Our findings unveiled the YBX3-mediated linking between BCAA metabolism and BAT differentiation and thermogenesis, providing a potential therapeutic approach against obesity and related metabolic dysfunction.

## 2 Materials and methods

### 2.1 Animals

Eight-week-old C57BL/6J wild-type male mice were purchased from Beijing Vital River Laboratory Animal Technology Co., Ltd (China). All animals were kept in the specific pathogen-free animal facility in the Laboratory Animal Research Center of Xiangya Hospital, Central South University, on a 12-hour dark/light cycle with ad libitum chow diet (ND) and water. For the diet-induced obesity model, mice were fed a 60% kcal high-fat diet (HFD, Research Diets, D12492) for four weeks. For CL-316,243 treatment, mice were daily intraperitoneal injected with 1 mg/kg CL-316,243 (MedChem Express, 138908-40-4) and housed at 30 °C for seven days. At 24 h post-last injection, fat tissues were collected for followed experiments. For cold exposure experiments, mice were single-housed without bedding at 6 °C with ad libitum access to food and water. Core body temperature was monitored hourly. At the end of the experiment, fat tissues were collected for followed experiments. The experimental animal protocols were approved by the Animal Ethics Committee according to the Guidelines for the Care and Use of Laboratory Animals at Xiangya Hospital of Central South University (Approval number: 202311116).

### 2.2 Intra-BAT injection of adeno-associated virus

Recombinant adeno-associated serotype 9 viruses with *Fabp4* promoter for *Ybx3* overexpression (OE, AAV-*Ybx3*) or *Ybx3* knockdown (KD, AAV-sh*Ybx3*) in adipocytes and control AAV (AAV-GFP and AAV-shNC) were purchased from Hanbio Biotechnology Co., Ltd. (China). AAV-*Ybx3* and AAV-sh*Ybx3* were injected in situ into the BAT of 2-month-old mice as previously described [5] at 1.5 × 10^10^ vg per side. The control groups were injected with control AAV. Mice were used for further experiments four weeks after injection.

### 2.3 Indirect calorimetry experiments

Indirect calorimetry experiments were conducted with the Promethion metabolic cage system (Sable Systems International, USA). Mice in the metabolic cage system were single-housed with a 12 h dark/light cycle and ad libitum access to food and water. For cold exposure experiment, mice were subsequently exposed to room temperature (22 °C, 48 h) and cold exposure (4 °C, 24 h). For CL-316,243 experiment, mice were housed at 30 °C for three days, followed by acute intraperitoneal injection of 1 mg/kg CL-316,243, and were kept at 30 °C for another 24 h measurement. Oxygen consumption and carbon dioxide production were continuously monitored to calculate the energy expenditure. Activities and food intake were collected continuously. All parameters were binned hourly over room temperature or cold exposure period using Sable Systems Macro Interpreter. The covariance (ANCOVA) analysis for light, dark, and total was performed using CalR (Version 1.3) [16].

### 2.4 Glucose tolerance test (GTT) and insulin tolerance test (ITT)

Mice were fasted for 6 h (ITT) or overnight (GTT) and treated with an intraperitoneal injection of insulin (0.75 U/kg, ITT) or glucose (1 g/kg, GTT). Tail venous blood was collected at the indicated time after glucose or insulin injection, and blood glucose levels were measured using the glucometer.

### 2.5 BCAA tolerance test

Eight-week-old male mice were gavaged with a single bolus of BCAA (500 mg/kg, Val: Leu: Ile = 1: 1.5: 0.8) and were exposed to cold at 12 °C under the fasting condition. Tail venous blood was collected at the indicated time points, and total plasma BCAA levels were measured using a commercial kit (Abcam, ab83374).

### 2.6 Serum parameters measurement

Serum parameters including serum triglyceride (TG, Elabscience, E-BC-K261-M), total cholesterol (TC, Elabscience, E-BC-K109-S), high-density lipoprotein cholesterol (HDL, Elabscience, E-BC-K221-M), low-density lipoprotein cholesterol (LDL, Elabscience, E-BC-K205-M), free fatty acids (FFA, Elabscience, E-BC-K792-M), and BCAA (Abcam, ab83374) were measured using commercial kits according to manuals.

### 2.7 Serum amino acid measurement

Mice whole blood was centrifugated at 3000 g for 20 min to separate serum and blood cells. The serum amino acids were analyzed using high-performance liquid chromatography (Ultimate 3000, USA)-tandem mass spectrometry (API 3200 Q-TRAP, USA) (HPLC-MS) methods.

### 2.8 Cell culture, adipogenic differentiation, and treatment

HEK293T cells (Procell, CL-0005) and C3H10T1/2 preadipocytes (Procell, CL-0325) were cultured in DMEM supplemented with 10% FBS, 1% penicillin/streptomycin solution at 37 °C in a humidified incubator with 5% CO2. Brown adipocyte differentiation was induced using the modified protocol described previously [4]. Briefly, confluent C3H10T1/2 preadipocytes were incubated with culture medium supplemented with 0.5 mM IBMX (Sigma, I5879), 1 μM dexamethasone (Sigma, D4902), 850 nM insulin (Sigma, 91077C), 1 nM triiodothyronine (MedChem Express, HY-A0070A) and 1 μM rosiglitazone (MedChem Express, HY-17386) for two days. Then, cells were maintained in the culture medium supplemented with 850 nM insulin, 1 nM triiodothyronine, and 1 μM rosiglitazone for another two days. Differentiated brown adipocytes on day 4 were maintained in the culture medium until they were used for experiments. For cell treatment, differentiated brown adipocytes were treated with forskolin (MedChem Express, HY-15371, 10 μM) for 24 h.

### 2.9 Lentiviral packaging and infection

The pCDH lentiviral vector was used to perform *Ybx3* gain-of-function experiments. For lentiviral packaging, pCDH-*Ybx3* plasmid or pCDH vector were co-transfected with packaging plasmid pMD2.G and pSPAX2 into HEK293T cells using Hieff Trans® Liposomal Transfection Reagent (Yeasen). Cells were maintained in the culture medium supplemented with 1% BSA and 10 mM Hepes post-transfection, and the culture medium was harvested at 48 h post-transfection. C3H10T1/2 preadipocytes were infected with the harvested lentiviruses in the presence of 4 mg/mL polybrene for 48 h, followed by puromycin selection (MedChem Express, HY-K1057, 10 mg/ml). The selected cells with stable overexpression of *Ybx3* (*Ybx3*) and control (Vector)cells were maintained in the culture medium containing 5 mg/ml puromycin for further experiments.

### 2.10 Plasmid and siRNAs transfection

All transfections that occurred before or after induction of adipocyte differentiation were performed using Lipofectamine 3000 (Invitrogen) according to the manufacturer’s manuals, and transfected cells were used for further experiments 48 h post-transfection. siRNAs targeting *Ybx3* (si*Ybx3*), *Slc3a2* (si*Slc3a2*), or *Pparg* (si*Pparg*) were purchased from Ribobio, China.

### 2.11 Mitochondrial stress test and intracellular ATP measurement

The Seahorse Bioscience XFe96 FLUX Analyzer (Agilent) was used to measure the oxygen consumption rate (OCR) of mature brown adipocytes with *Ybx3* knockdown or overexpression and control cells. In brief, cells were seeded at a density of 5 × 10^3^ cells/well into 96-well plates and allowed for adherence to the bottom overnight. Cells were incubated with DMEM containing 25 mM d-glucose, 1 mM sodium pyruvate, and 2 mM l-glutamine for 1 h at 37 °C without CO_2_ before measurement. The mitochondrial stress test utilizes sequential injections of 4 mM oligomycin A (Glpbio), 4 mM FCCP (Sigma), and 1 mM rotenone (Sigma)/antimycin A (Glpbio). The parameters of OCR were automatically calculated by the WAVE desktop software (Agilent). All parameters were normalized to the total protein amount in each well using the BCA protein assay (Elabscience). The intracellular ATP levels were measured using the Enhanced ATP Assay Kit (Beyotime, S0026) according to the manufacturer’s instructions.

### 2.12 Measurement of mitochondrial DNA (mtDNA)/nuclear DNA (nDNA)

The total DNA was isolated from mature brown adipocytes. The ratio of mtDNA/nDNA was calculated using the copy number of mtDNA gene ND1 and 16S normalized against the nDNA gene Hk2. The primer sequences are listed in Supplementary Table 1.

### 2.13 RNA extraction and qPCR analysis

Total RNAs were extracted using AG RNAex Pro Reagent (Accurate Biotechnology (Hunan) Co., Ltd). Then, cDNA synthesis and qPCR assay were performed as previously reported [17]. The relative mRNA levels were calculated by the 2−^ΔΔ^Ct method using β-Actin as an internal control. The primers used for qPCR were listed in Supplemental Table 1.

### 2.14 mRNA stability assay

C3H10T1/2 preadipocytes were transfected with siNC and si*Ybx3* for 48 h and differentiated into brown adipocytes for four days. Differentiated adipocytes were treated with 10 mg/ml actinomycin D (MedChem Express, HY-17559) and harvested at indicated time points. The same amount of total RNA from each group at different times was used to conduct reverse transcription with random primers and qPCR. As previously reported, the mRNA half-life was calculated using the one-phased decay model [12].

### 2.15 RNA-seq analysis

C3H10T1/2 preadipocytes were transfected with siNC or si*Ybx3* for 48 h and then subjected to brown adipocyte differentiation. Total RNAs of differentiated adipocytes were extracted for commercial RNA-seq (Oebiotech, Shanghai, China). Gene set enrichment analysis (GSEA) was performed based on the Kyoto Encyclopedia of Genes and Genomes (KEGG) gene sets to show the most affected biological pathway between the two groups.

### 2.16 Immunoblot analysis

Immunoblot was conducted as previously described [18]. The antibodies used are YBX3 (Proteintech, 27785-1-AP), YBX3 (Invitrogen, 40–2800), UCP1 (Abcam, ab10983), PGC-1α (SCBT, sc-517380), PRDM16 (Abcam, ab106410), PPARγ (SCBT, sc-7273), FABP4(CST, 2120), SLC3A2 (Proteintech, 15193-1-AP), SLC7A5 (Proteintech, 28670-1-AP), p-CREB (CST, 9198), CREB (Proteintech, 12208-1-AP), p-PKA substrates (CST, 9621), ATGL (CST, 2138), p-HSL (CST, 4126), HSL (CST, 4107), p-P38 (CST, 4511), P38 (CST, 8690), p-ERK (CST, 4370), ERK (CST, 4695), Tubulin (Proteintech, 11224-1-AP).

### 2.17 Histochemistry and immunohistochemistry staining

H&E staining and immunohistochemical staining were performed as previously described [18]. The primary antibody used in immunohistochemistry staining is UCP1 antibody (Abcam, ab10983). The area of adipocytes was calculated using Fiji.

### 2.18 Statistical analysis

All experiments were performed at least three times. The data are expressed as mean ± SD or mean ± SEM as indicated. Two-tailed Student’s t-test or Welch’s t-test was used to compare the two groups. When comparing the difference between multiple groups, one-way ANOVA with Tukey multiple comparison tests, two-way ANOVA with Dunnett multiple comparison test, or Bonferroni multiple comparison test was applied. The Analysis of Covariance (ANCOVA) test analyzed metabolic cage parameters with body weight as a covariate. Statistical differences were supposed to be significant when *P* < 0.05. The analysis was conducted using GraphPad 8.0 software and CarlR (https://calrapp.org/). Significance in all figures is denoted as follows: ∗*p* < 0.05, ∗∗*p* < 0.01, ∗∗∗*p* < 0.001, ∗∗∗∗*p* < 0.0001.

## 3 Result

### 3.1 YBX3 is a BAT-enriched RBP responding to ambient temperature and cAMP signaling

To identify putative RBPs involved in brown adipocyte differentiation and thermogenesis, 1,912 canonical mouse RBPs annotated in EuRBPDB [19] were included in the subsequent investigation. Many of these RBPs have not been previously linked to adipose biology. We used multiple public GEO datasets (GSE181123, GSE222424, and GSE29897) to explore the RBPs expression patterns during cold challenge or brown adipocyte differentiation (Figure 1A–C). By integrating the analysis of differentially expressed RBPs across the three datasets, we identified 25 candidate RBPs that were significantly upregulated during cold challenge and brown adipogenesis (Figure 1D). We next investigated the expression patterns of the selected candidate RBPs across various tissues using the BioGPS database [20]. The expression of *Ybx3* is among the highest in multiple tissues compared to other RBPs, including in BAT, WAT, and skeletal muscle (Figure 1E). We thus took a deep dive into the role of *Ybx3* in adipose biology. Among the different adipose depots (BAT, visceral adipose tissue (VAT), and subcutaneous adipose tissue (SAT)), YBX3 is predominantly expressed in BAT (Figure 1F,G). Next, we investigated the BAT *Ybx3* expression alteration in response to ambient temperature change. The BAT *Ucp1* expression significantly increased after a seven-day cold challenge and went down upon thermoneutrality (TN) exposure, reflecting the induction of thermogenesis and whitening, respectively (Figure 1H–K). Meanwhile, the *Ybx3* expression synchronously increased or decreased in response to cold or thermoneutrality exposure (Figure 1H–K), suggesting the potential regulator role of *Ybx3* in BAT thermogenesis. β3-adrenergic signaling plays a master role in cold-induced adaptive thermogenesis. We thereby treated mice with β3-adrenergic receptor agonist CL-31,624 (CL) at TN for seven days to determine whether β3-adrenergic signaling affects the expression of *Ybx3*. The results showed that *Ybx3* expression was reciprocally upregulated with *Ucp1* expression upon CL treatment (Figure 1L and M). Together, our data identified YBX3 as a BAT-enriched RBP in response to ambient temperature and β3-adrenergic signaling activation.

**Figure 1:**
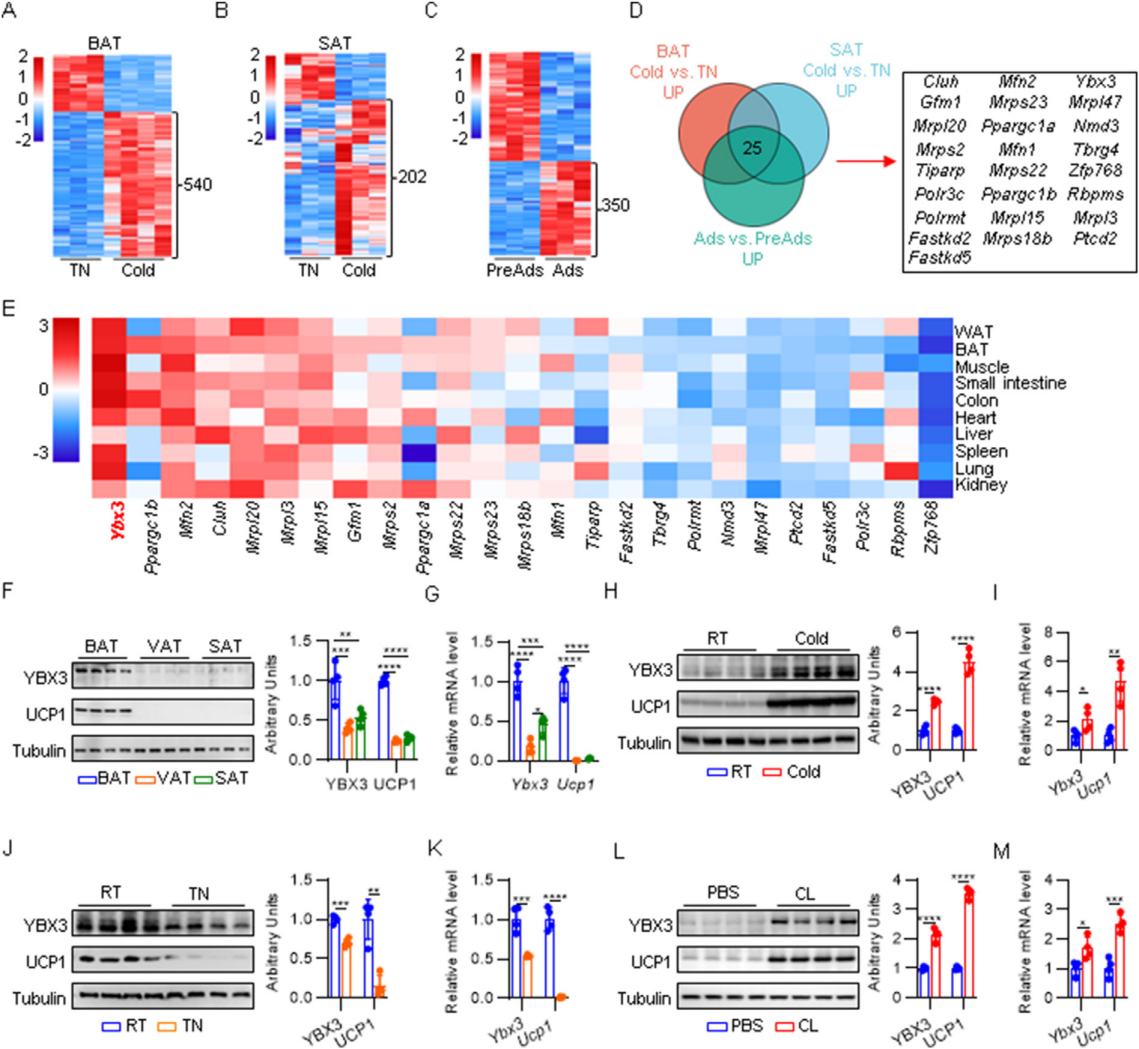
**YBX3 is a brown adipocyte-enriched RBP responding to ambient temperature and adrenergic signaling**. (A) Heatmap of differentially expressed RBPs genes in the BAT from mice exposed to cold and thermoneutrality (TN) (*n* = 3–4, *p* < 0.05, |log_2_FC| >1.5). (B) Heatmap of differentially expressed RBPs genes in the SAT from mice exposed to cold and TN (*n* = 3, *p* < 0.05, |log_2_FC| >1.5). (C) Heatmap of differentially expressed RBPs genes in mature adipocytes versus preadipocytes (*n* = 3, *p* < 0.05, |log_2_FC| >1.3). (D) Venn diagram shows 25 shared upregulated RBPs from (A), (B), (C). (E) Heatmap of RBPs genes expression patterns across different tissues. (F) Immunoblot of YBX3 and UCP1 in BAT, VAT, and SAT (left) and quantification (right) (*n* = 4). (G) mRNA levels of *Ybx3* and *Ucp1* in BAT, VAT and SAT. (*n* = 4). (H) Immunoblot of YBX3 and UCP1in BAT of mice exposed to room temperature (RT) or cold (left) and quantification (right) (*n* = 4). (I) mRNA levels of *Ybx3* and *Ucp1* in BAT of mice exposed to RT or cold (*n* = 4). (J) Immunoblot of YBX3 and UCP1 in BAT of mice exposed to RT or TN (left) and quantification (right) (*n* = 4). (K) mRNA levels of *Ybx3* and *Ucp1* in the BAT of mice exposed to RT or TN (*n* = 4). (L) Immunoblot of YBX3 and UCP1 in the BAT of mice treated with CL-316,243 (CL) or PBS (left) and quantification (right) (*n* = 4). (M) mRNA levels of *Ybx3* and *Ucp1* in the BAT of mice treated with CL or PBS (*n* = 4). Data are shown as the mean ± SD. ∗*P* < 0.05, ∗∗*P* < 0.01, ∗∗∗*P* < 0.001, ∗∗∗∗*P* < 0.0001 by one-way ANOVA with Tukey multiple comparison tests (F–G) or two-tailed Student’s t-test (H–M). (For interpretation of the references to colour in this figure legend, the reader is referred to the Web version of this article.)

### 3.2 YBX3 is essential for brown adipocyte differentiation and thermogenesis *in vitro*

Like other YBX members, YBX3 has been reported to participate in cell proliferation and differentiation [21]. We therefore asked whether YBX3 plays a role in brown adipocyte differentiation and thermogenesis. We first queried one public single-nucleus RNA-seq dataset to analyze single-cell *Ybx3* expression patterns in BAT [22]. The expression analysis showed that *Ybx3* is mainly expressed in adipocytes among the annotated cell types (Figure S1A and B). Of note, adipocytes with higher *Ybx3* expression showed higher expression levels of thermogenic genes, such as *Ucp1* and *Ppargc1a* (Figure S1B), indicating a potential correlation between *Ybx3* and the thermogenic genes. Cell fractions analysis confirmed that the YBX3 protein is enriched in mature adipocytes rather than SVF fraction cells (Figure S1C). Next, we examined the temporal expression pattern of *Ybx3* during brown adipocyte differentiation. Upon adipogenic stimulation, *Ybx3* expression robustly increased, accompanied by high levels of adipogenic transcription factors (*Cebpb*, *Pparg,* and *Prdm16*), pan-adipocyte makers (*Adipoq* and *Fabp4*), and brown adipocyte markers (*Ppargc1a*, *Ucp1*, *Cidea,* and *Dio2*) (Figure S1D). The mature brown adipocytes showed higher protein levels of YBX3 and brown adipocyte markers than preadipocytes (Figure S1E). Consistent with the effects of β3-adrenergic treatment *in vivo*, the cAMP-inducer forskolin enhanced the *Ybx3* expression in mature brown adipocytes at both protein and mRNA levels (Figure S1F and G). These results implied that YBX3 is a brown adipocyte-enriched RBP, which may play a role in brown adipocyte differentiation and thermogenesis. To determine the function of *Ybx3* in brown adipocyte differentiation, we knocked down or overexpressed *Ybx3* in C3H10T1/2 preadipocytes before inducing brown adipocyte differentiation. Remarkably, *Ybx3* knockdown (si*Ybx3*) in preadipocytes decreased the expression of adipogenic transcription factors, pan-adipocyte makers, and brown adipocyte markers compared with control cells (siNC) post-differentiation (Figure 2A and B). Conversely, lentiviral overexpression of *Ybx3* promotes brown adipocyte differentiation as indicated by enhanced expression of brown adipogenic-related genes (Figure 2C and D). These data suggested that *Ybx3* is essential for brown adipocyte differentiation *in vitro*.

**Figure 2:**
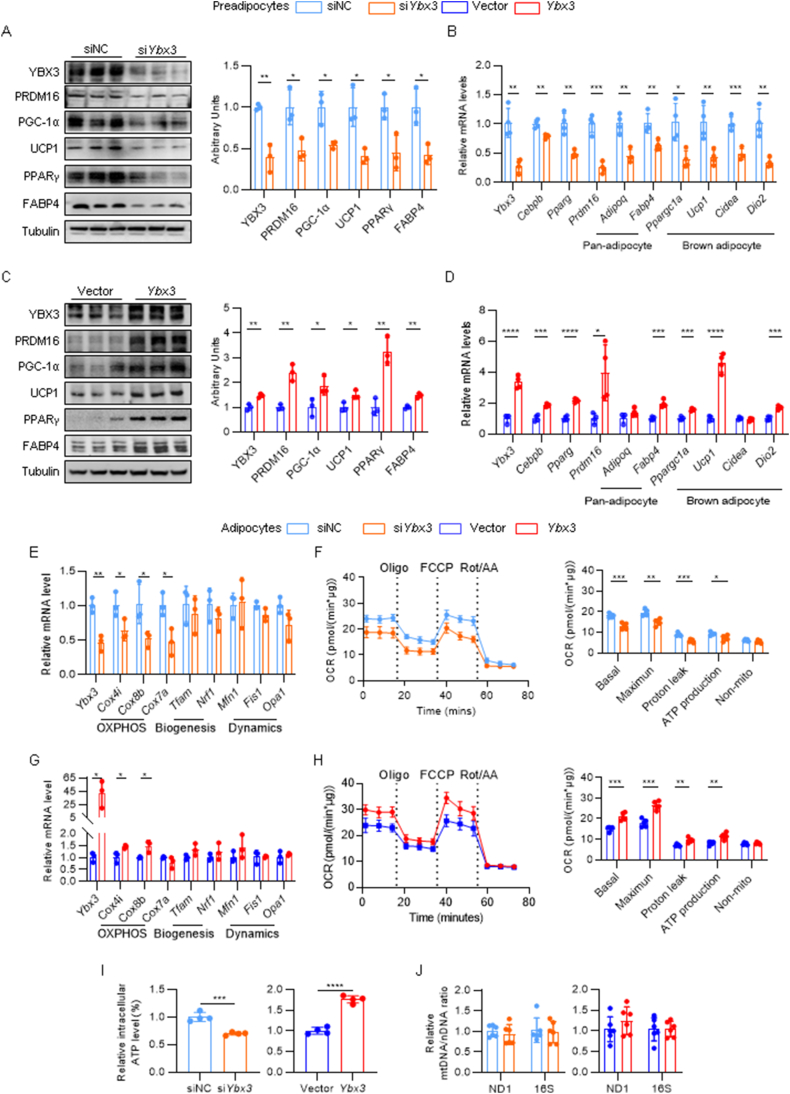
**YBX3 is essential for brown adipocyte differentiation and thermogenesis*****in vitro***. (A, C) Immunoblots of YBX3, adipogenic, and thermogenic proteins in *Ybx3* knockdown (A) or overexpressed (C) adipocytes and control cells (left) and quantification (right) (*n* = 3). (B, D) mRNA levels of *Ybx3*, adipogenic, and thermogenic genes in *Ybx3* knockdown (B) or overexpressed (D) adipocytes and control cells (*n* = 4). (E, G) mRNA level of genes related to mitochondrial OXPHOS, biogenesis, and dynamics in mature brown adipocytes with *Ybx3* knockdown or overexpression and control cells (*n* = 3). (F, H) OCR curve of mature brown adipocytes with *Ybx3* knockdown (F) or overexpression (H) and control cells (left), and quantification (right) (*n* = 5). Rot, rotenone; AA, antimycin. (I) Intracellular ATP levels of mature brown adipocytes with *Ybx3* knockdown or *Ybx3* overexpression and control cells (*n* = 3). (J) The mtDNA/nDNA ratio of mature brown adipocytes with *Ybx3* knockdown or *Ybx3* overexpression and control cells (*n* = 6). Data are shown as the mean ± SD or mean ± SEM (F and H). ∗*P* < 0.05, ∗∗*P* < 0.01, ∗∗∗*P* < 0.001, ∗∗∗∗*P* < 0.0001 by two-tailed Student’s t-test (A–I). (For interpretation of the references to colour in this figure legend, the reader is referred to the Web version of this article.)

We further performed RNA-seq of *Ybx3* knockdown preadipocytes versus control cells by the end of differentiation. GSEA analysis of the KEGG pathway showed that the oxidative phosphorylation (OXPHOS) pathway topped the most affected pathway upon *Ybx3* knockdown (Figure S2A and B). It is well-established that uncoupling of mitochondrial OXPHOS is the major contributor to BAT heat production [2]. We thus speculated that *Ybx3* knockdown impairs mitochondrial OXPHOS and leads to thermogenic failure. In mature brown adipocytes, loss of *Ybx3* reduces thermogenic gene expressions, while gain of *Ybx3* enhances them (Figure S2C–F). Consistent with the RNA-seq data, mature brown adipocytes with *Ybx3* knockdown showed decreased mRNA levels of OXPHOS core genes (Figure 2E). Mitochondrial stress test showed that *Ybx3* knockdown significantly decreases basal and maximum respiration, ATP production, and proton leak without affecting non-mitochondrial OCR (Figure 2F). Furthermore, mature brown adipocytes with *Ybx3* knockdown showed decreased intracellular ATP levels (Figure 2I). Conversely, *Ybx3* overexpression enhanced mitochondrial OXPHOS as indicated by OXPHOS gene expressions (Figure 2G), mitochondrial respiration (Figure 2H), and intracellular ATP levels (Figure 2I). We further asked whether YBX3 affects mitochondrial biogenesis and dynamics. Compared to control cells, neither loss nor gain of *Ybx3* affects the expression of essential genes related to mitochondrial biogenesis (*Tfam* and *Nrf1*) and dynamics (*Mfn1*, *Fis1,* and *Opa1*) [23] (Figure 2E,G). The mtDNA/nDNA ratio analysis also showed little effect of *Ybx3* knockdown or overexpression on mtDNA copies (Figure 2J). Together, these results demonstrated that YBX3 is essential for brown adipocyte differentiation and thermogenesis *in vitro*.

### 3.3 BAT-specific loss of *Ybx3* impairs thermogenesis and exacerbates diet-induced obesity

To investigate the *in vivo* role of *Ybx3* in the regulation of BAT thermogenesis, we generated BAT-specific *Ybx3* knockdown (KD) mice by in situ injection of *Fabp4* promoter-driven AAV expressing short hairpin RNA targeting *Ybx3* (AAV-sh*Ybx3*) or control AAV (AAV-shNC) into BAT. Four weeks after injection, we thoroughly examined the expression of *Ybx3* across multiple tissues. The results showed that AAV-sh*Ybx3* knocked down *Ybx3* expression in brown adipocytes without affecting other tissues, such as SAT, VAT, and skeletal muscle (Figure S3A–C). When exposed to acute cold stress, the KD mice failed to maintain core body temperature compared to control mice (Figure 3A). Consistently, the KD mice showed a significant decrease in oxygen consumption and energy expenditure during room temperature and cold exposure (Figure 3B, Figure S3D) without affecting physical activity or food intake (Figure S3E and F). To exclude the effect of other thermogenic processes, such as shivering thermogenesis of skeletal muscle under cold exposure, KD mice and control mice were kept at 30 °C for three days and treated with acute intraperitoneal injection of CL to activate BAT thermogenesis. The administration of CL resulted in a lower core temperature elevation in the KD mice compared to the control mice (Figure 3C). Meanwhile, the KD and control mice showed equal oxygen consumption and energy expenditure when kept at 30 °C. However, control mice showed much higher oxygen consumption and energy expenditure upon CL treatment than KD mice (Figure 3D, Figure S3G) without affecting activity or food intake (Figure S3H–I). These results indicated impaired CL-triggered BAT thermogenic response in KD mice. The reduced expression of thermogenic genes in BAT, as indicated by *Ppargc1a*, *Ucp1*, *Cidea*, and *Dio2*, also reflected the molecular-level dysfunction of thermogenesis in KD mice (Figure 3E–G). However, SAT thermogenic gene expressions were unaffected (Figure S3J). Dampened thermogenesis and decreased energy expenditure could impair glucose homeostasis and increase susceptibility to diet-induced obesity [5]. Thus, we assessed whether loss of *Ybx3* leads to metabolic dysfunction. When fed a ND, the body weight gain, fat mass, and adipocyte size of different adipose depots in the KD mice resembled those in the control mice (Figure S3K–N). Next, we fed the KD and control mice a 60% kcal HFD to investigate the impact of *Ybx3* knockdown in diet-induced obesity and glucose dysfunction. With HFD feeding, KD mice showed higher body weight gain (Figure 3H), more severe glucose intolerance, and insulin resistance (Figure 3I and J) than control mice. Meanwhile, the fat mass, liver mass, adipocyte size, and hepatic lipid droplets significantly increased in KD mice (Figure 3K-N). The serum TG, TC, LDL, and FFA levels also increased in *Ybx3* knockdown mice (Figure S3O). These data showed that BAT-specific loss of *Ybx3* impairs thermogenesis and exacerbates diet-induced obesity.

**Figure 3:**
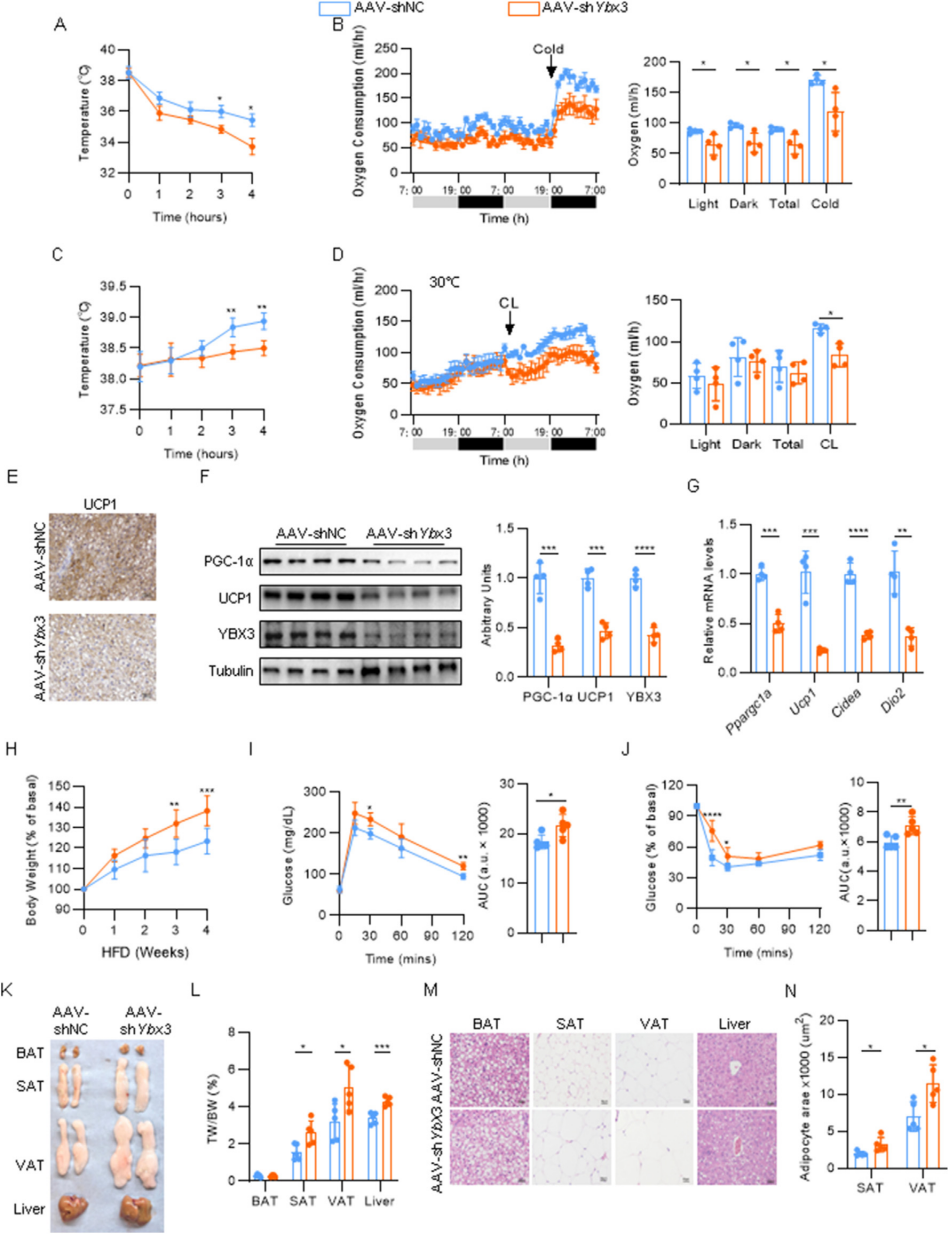
**BAT-specific loss of *Ybx3* impairs thermogenesis and exacerbates diet-induced obesity**. (A) Core temperature under cold exposure (*n* = 4). (B) Hourly curve of oxygen consumption (left) and quantification (right) under ND feeding (*n* = 4). (C) Core temperature after CL treatment (*n* = 5). (D) Hourly curve of oxygen consumption (left) and quantification (right) under CL treatment (*n* = 4). (E) IHC staining of UCP1 in the BAT after acute cold exposure (*n* = 4, Bar = 50 μm). (F) Immunoblots of PGC-1α, UCP1, and YBX3 in the BAT after acute cold exposure (left) and quantification (right) (*n* = 4). (G) mRNA levels of thermogenic genes in the BAT after acute cold exposure (*n* = 4). (H) Body weight gain curve under HFD feeding (*n* = 5). (I–J) GTT (I) and ITT (J) under HFD feeding (*n* = 5). (K) Gross picture of BAT, SAT, VAT, and liver. (L) The ratio of adipose tissue and liver weight to body weight (*n* = 5). (M) H&E staining of BAT, SAT, VAT, and liver (*n* = 5, Bar = 50 μm). (N) The adipocyte area quantification of SAT and VAT (*n* = 5). Data are shown as the mean ± SD or mean ± SEM (B and D). ∗*P* < 0.05, ∗∗*P* < 0.01, ∗∗∗*P* < 0.001, ∗∗∗∗*P* < 0.0001 by two-way ANOVA with Bonferroni multiple comparison test (A, C, H-J), ANCOVA with body weight as covariant (B, D) or two-tailed Student’s t-test (F-G, I-J, L, N).

### 3.4 YBX3 stabilizes *Slc3a2* mRNA to facilitate BCAA transport and fuel thermogenic adipocyte differentiation

As a canonical RBP, YBX3 has an established role in controlling mRNA translation and decay [24]. Two independent studies demonstrated that YBX3 enhances the mRNA stability of *Slc3a2* or *Slc7a5*, which encodes the heterodimeric SLC7A5/SLC3A2 BCAA transporter and regulates BCAA transport and maintain intracellular BCAA levels [24,25]. Previous studies indicated that BCAA catabolism fuels adipocyte differentiation and mitochondrial OXPHOS, facilitating cold-induced BAT thermogenesis [26,27]. We thus asked whether the YBX3-SLC3A2-BCAA axis is involved in brown adipocyte differentiation and thermogenesis. The *Ybx3* knockdown brown adipocytes showed decreased mRNA and protein levels of *Slc3a2* but not *Slc7a5* (Figure 4A and B). Using actinomycin D to stop mRNA transcription, *Ybx3* knockdown brown adipocytes showed a significantly accelerated mRNA decay rate of *Slc3a2,* whose mRNA half-life decreased from 35 h to 3 h. In contrast, the half-life of *Slc7a5* mRNA was not affected (Figure 4C). In vivo, loss of *Ybx3* reduced the level of *Slc3a2* but not *Slc7a5* in the BAT (Figure 4D). These results confirmed the YBX3-mediated post-transcriptional regulation of *Slc3a2* in BAT. Cold-induced BAT activation promotes systemic BCAA clearance to fuel thermogenesis [26]. We collected blood serum from KD and control mice after acute cold exposure and analyzed the serum amino acid levels using HPLC/MS. The serum levels of valine (Val) and leucine (Leu) were significantly increased in KD mice compared to control mice (Figure 4E). Serum BCAA analysis using commercial kits confirmed higher circulation BCAAs levels in HFD-fed KD mice (Figure 4F). We next conducted the BCAA tolerance test at 12 °C. Upon oral BCAA challenge, the KD mice exhibited elevated serum BCAA levels and a slower rate of BCAA clearance in circulation than control mice (Figure 4G). These results indicated impairment of the YBX3-SLC3A2 axis in BAT disturbed systemic BCAA clearance.

**Figure 4:**
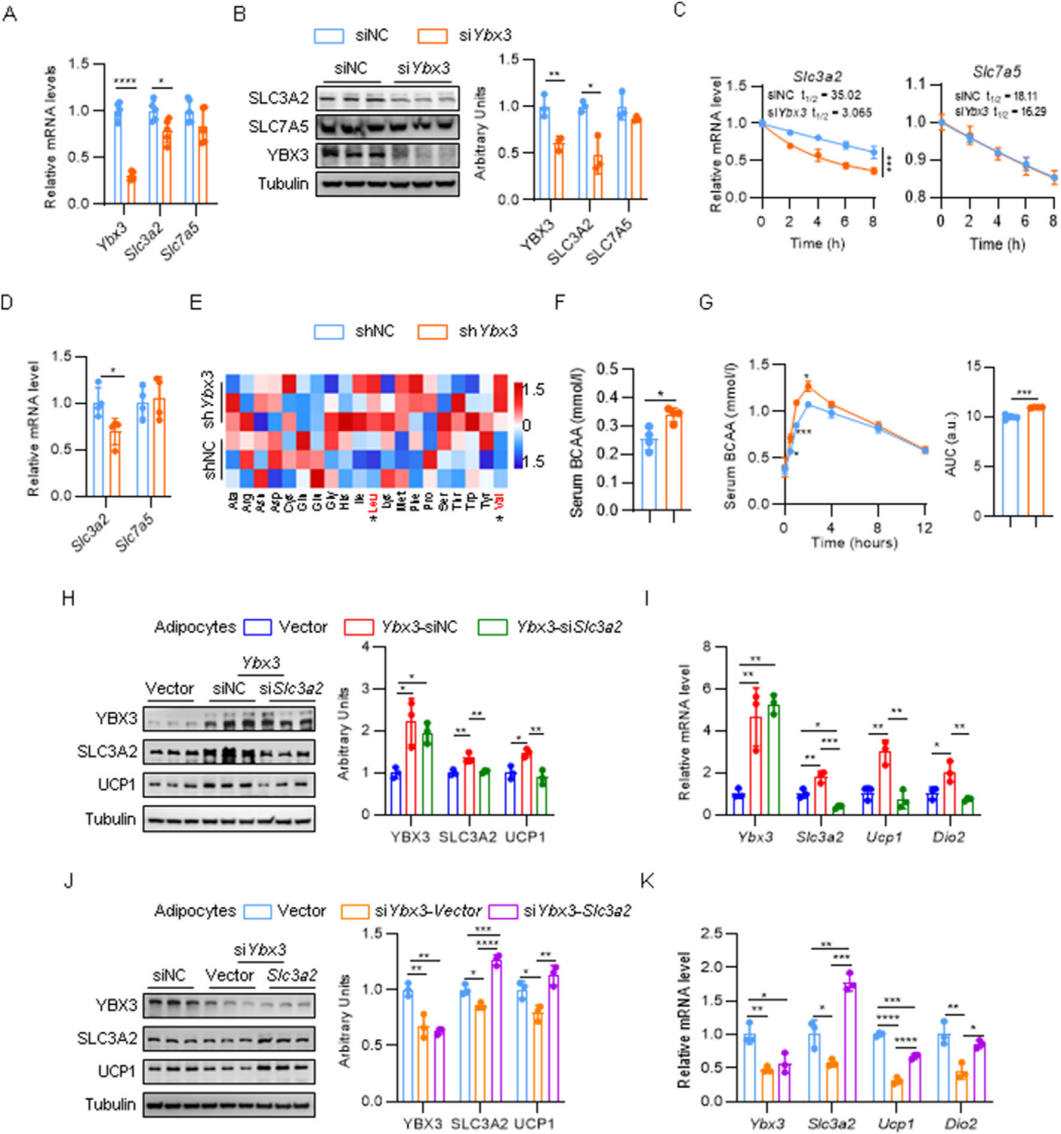
**YBX3 stabilizes *Slc3a2* mRNA to facilitate BCAA influx and fuel brown adipocyte differentiation and thermogenesis**. (A) mRNA level of *Ybx3*, *Slc3a2* and *Slc7a5* in *Ybx3* knockdown brown adipocytes and control cells (*n* = 5). (B) Immunoblots of YBX3, SLC3A2, and SLC7A5 in *Ybx3* knockdown brown adipocytes and control cells (left) and quantification (right) (*n* = 3). (C) mRNA level of *Slc3a2* and *Slc7a5* in *Ybx3* knockdown brown adipocytes and control cells at indicated time-points after actinomycin D (10 mg/ml) treatment (*n* = 3). (D) mRNA level of *Slc3a2* and *Slc7a5* in BAT under ND feeding (*n* = 4). (E) Heatmap of serum amino acid levels after acute cold exposure under ND feeding (*n* = 3). (F) Serum BCAA levels under HFD feeding (*n* = 4). (G) Serum BCAA level curve during BCAA tolerance test (left) and quantification (right) under ND feeding (*n* = 4). (H) Immunoblots of YBX3, SLC3A2, and UCP1 in brown adipocytes with indicated treatment (left) and quantification (right) (*n* = 3). (I) mRNA level of *Ybx3*, *Slc3a2,* and thermogenic genes in mature brown adipocytes with indicated treatment (*n* = 4). (J) Immunoblots of YBX3, SLC3A2, and UCP1 in mature brown adipocytes with indicated treatment (left) and quantification (right) (*n* = 3). (K) mRNA level of *Ybx3*, *Slc3a2,* and thermogenic genes in mature brown adipocytes with indicated treatment (*n* = 4). Data are shown as the mean ± SD. ∗*P* < 0.05, ∗∗*P* < 0.01, ∗∗∗*P* < 0.001, ∗∗∗∗*P* < 0.0001 by two-tailed Student’s t-test (A-B, D, F-G), two-way ANOVA with Bonferroni multiple comparison test (C, G), one-way ANOVA with Tukey multiple comparison test (H–K). (For interpretation of the references to colour in this figure legend, the reader is referred to the Web version of this article.)

We then investigated how YBX3-SLC3A2 axis-mediated BCAA metabolism affects brown adipocyte differentiation and thermogenesis *in vitro*. We first conducted loss- and gain-of-function experiments of *Slc3a2* in both preadipocyte (Figure S4A–D) and mature adipocyte stages (Figure S4E–H) with or without BCAAs in the culture medium. In the presence of BCAAs, *Slc3a2* knockdown impaired brown adipocyte differentiation and thermogenesis, while *Slc3a2* overexpression showed a reversed effect (Figure S4A–H). However, BCAA deprivation abolished the impact of *Slc3a2* knockdown and *Slc3a2* overexpression on brown adipogenic and thermogenic gene expressions (Figure S4A–H). In short, *Slc3a2* enables brown adipocyte differentiation and thermogenesis in a BCAAs-dependent manner. We then performed the loss-of-function experiment of *Slc3a2* in *Ybx3* overexpressed preadipocytes (Figure S4I and J) and mature adipocytes (Figure 4H and I). Remarkedly, loss of *Slc3a2* diminished the promoting effects of *Ybx3* overexpression on brown adipogenic (Figure S4I and J) and thermogenic (Figure 4H and I) gene expressions. We next explored whether overexpression of *Slc3a2* could rescue brown adipogenesis and thermogenic program impaired by *Ybx3* deficiency. Ectopic *Slc3a2* overexpression counteracted the accelerated mRNA decay of *Slc3a2* in the absence of *Ybx3* and significantly promoted brown adipogenic (Figure S4K–L) and thermogenic (Figure 4J and K) gene expressions. These results suggested that YBX3 could enhance the mRNA stability of *Slc3a2*, which facilitates BCAA influx and fuels brown adipocyte differentiation and BAT thermogenesis.

### 3.5 Loss of *Ybx3* disturbs PPARγ-dependent brown adipocyte thermogenesis and BCAA catabolism

Next, we explored whether YBX3 transcriptionally regulates the thermogenic genes. Given the predominant effects of β3-adrenergic signaling on the transcriptional regulation of thermogenic genes, we first analyzed whether YBX3 affects the multiple downstream signaling, including PKA/CREB pathway, PKA/HSL pathway, and MAPK pathway. The results showed that neither knockdown nor overexpression of *Ybx3* affects the phosphorylation of PKA substrates, CREB, HSL, p38, and ERK. The protein level of ATGL was not affected by *Ybx3* knockdown or overexpression (Figure S5A and B). We further tested the mRNA stability of *Pparg*, *Prdm16*, *Ppargc1a*, *Ucp1,* and *Dio2*, which were downregulated in *Ybx3* knockdown adipocytes. Of interest, the mRNA half-life of *Pparg* significantly decreased upon *Ybx3* knockdown while other genes showed no difference (Figure 5A). Given the well-established regulatory role of PPARγ in brown adipogenesis and thermogenesis [7], we proposed that YBX3 regulates adipogenic and thermogenic genes' transcription through stabilizing *Pparg* mRNA. We performed the loss-of-function experiment of *Pparg* in *Ybx3* overexpressed preadipocyte and mature adipocyte in the absence of PPARγ agonist rosiglitazone. We found that loss of *Pparg* abolished the enhanced brown adipogenic and thermogenic gene expression in *Ybx3* overexpressed adipocytes (Figure 5B–E), supporting that YBX3 regulates brown adipogenic and thermogenic genes in a PPARγ-dependent manner. In addition, PPARγ has been reported as a major regulator of BCAA catabolism [28]. We further investigated the involvement of the YBX3- PPARγ axis in BCAA metabolism. In line with previous studies, the expression of genes related to BCAA metabolism robustly increased during brown adipocyte differentiation (Figure S5C) [29], synchronizing with the increased *Ybx3* expression (Figure S1D and E). Loss of *Ybx3 in vitro* and *in vivo* simultaneously reduced the expression of a series of genes that encode BCAA catabolic enzymes (Figure S5D–F), suggesting dampened BCAA oxidation upon *Ybx3* deficiency. Of note, *Pparg* knockdown abolished the increased expression of BCAA catabolic enzymes in *Ybx3* overexpressed preadipocytes and mature adipocytes, such as *Bckdhb*, the BCAA catabolic rate-limiting enzyme (Figure 5F and G). We further tested the effects of *Pparg* knockdown on BCAA uptake. The results showed that the loss of *Pparg* does not affect the expression of *Slc3a2* at the protein or mRNA levels (Figure S5G and H). However, the culture medium of cells lacking *Pparg* showed a higher concentration of BCAAs than control cells (Figure S5I). Given the pivotal role of *Pparg* in BCAA catabolism, the excess BCAAs may result from decreased BCAA oxidation. These results indicated a PPARγ-dependent regulation of BCAA catabolism and thermogenesis by YBX3. Moreover, we found that glucose and fatty acid metabolism-related genes were partially down-regulated by *Ybx3* knockdown (Figure S5J). These changes may result from *Pparg* deficiency. Thus, we could not exclude the potential contribution of YBX3-mediated glucose and fatty acid metabolism in BAT thermogenesis.

**Figure 5:**
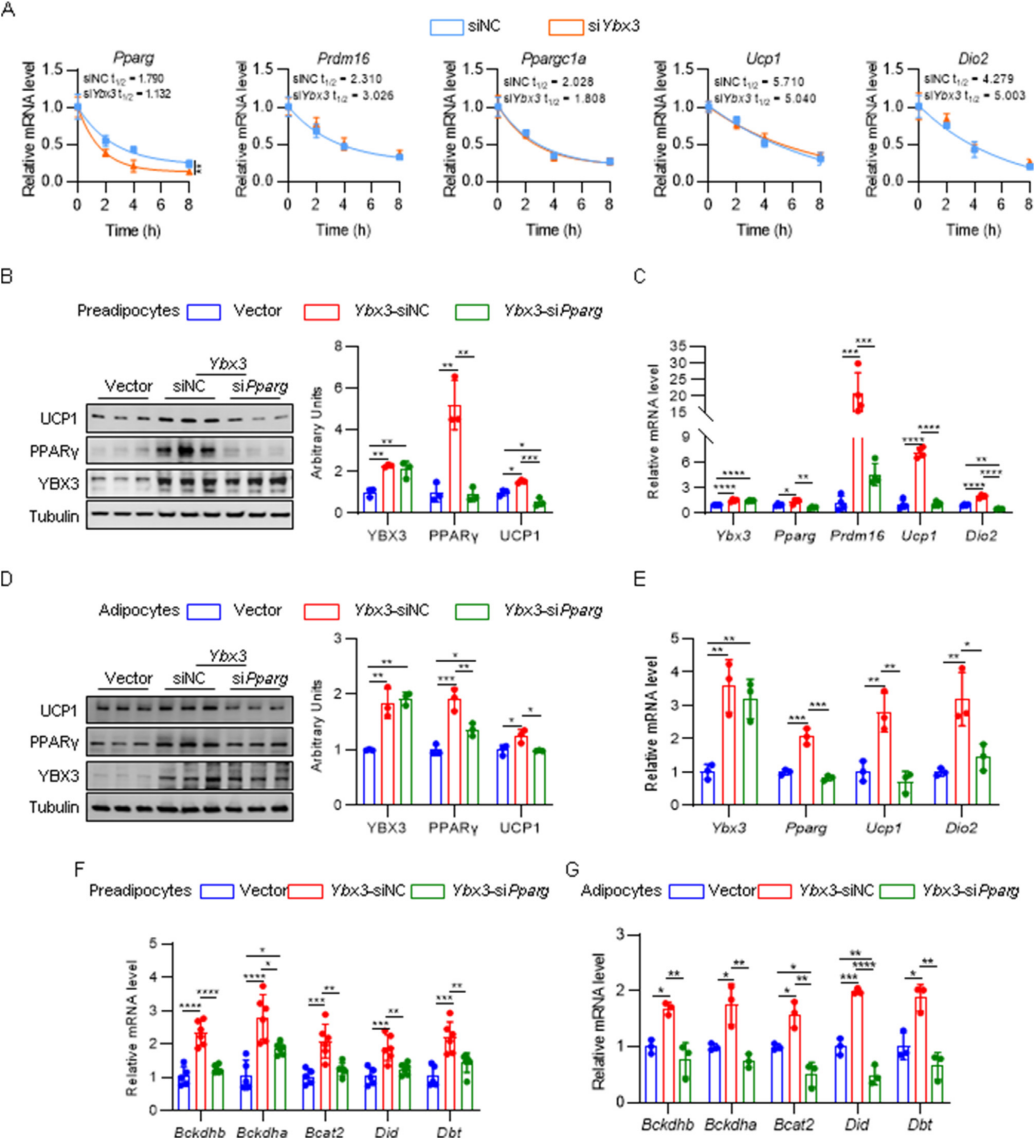
**Loss of *Ybx3* disturbs PPARγ-dependent brown adipocyte thermogenesis and BCAA catabolism**. (A) mRNA level of *Pparg* and thermogenic genes in *Ybx3* knockdown brown adipocytes and control cells at indicated time-points after actinomycin D treatment (*n* = 3). (B) Immunoblots of YBX3, PPARγ, and UCP1 in brown adipocytes with indicated treatment (left) and quantification (right) (*n* = 3). (C) mRNA level of *Ybx3*, *Pparg*, and thermogenic genes in brown adipocytes with indicated treatment (*n* = 4). (D) Immunoblots of YBX3, PPARγ, and UCP1 in mature brown adipocytes with indicated treatment (left) and quantification (right) (*n* = 3). (E) mRNA level of *Ybx3*, *Pparg*, and thermogenic genes in mature brown adipocytes with indicated treatment (*n* = 4). (F) mRNA level of BCAA catabolic genes in brown adipocytes with indicated treatment (*n* = 5–6). (G) mRNA level of BCAA catabolic genes in mature brown adipocytes with indicated treatment (*n* = 3). Data are shown as the mean ± SD. ∗*P* < 0.05, ∗∗*P* < 0.01, ∗∗∗*P* < 0.001, ∗∗∗∗*P* < 0.0001 by two-way ANOVA (A), one-way ANOVA with Tukey multiple comparison test (B–G). (For interpretation of the references to colour in this figure legend, the reader is referred to the Web version of this article.)

### 3.6 BAT-specific gain of *Ybx3* prompts thermogenesis to protect against diet-induced metabolic dysregulation

Having observed the critical role of *Ybx3* in brown adipocyte differentiation and thermogenesis, we asked whether *Ybx3* overexpression confers protection against diet-induced obesity and metabolic dysregulation. We generated BAT-specific *Ybx3* overexpression mice (OE) by in situ injection of *Fabp4* promoter-driven AAV expressing *Ybx3* (AAV-*Ybx3*) and control AAV (AAV-GFP) into BAT. Four weeks after injection, the efficiency and specificity of *Ybx3* overexpression were examined across multiple tissues (Figure S6A–C). In contrast to the thermogenic dysfunction phenotype in KD mice, OE mice showed better cold tolerance under acute cold exposure (Figure 6A and B). The OE mice also showed significantly increased oxygen consumption and energy expenditure increase across room temperature and cold stimulation period (Figure 6C and D) without affecting physical activity or food intake (Figure S6D and E). The thermogenic gene expressions measured by immunohistochemistry, immunoblots, and qPCR supported the boosted thermogenic program in BAT of OE mice (Figure 6E–G), while SAT thermogenic gene expressions were unaffected (Figure S6F). The mRNA and protein levels of *Slc3a2* also increased upon *Ybx3* overexpression *in vivo* (Figure 6F and G). Next, we examined the metabolic phenotypes of OE and control mice under ND or HFD conditions. When fed a ND, we observed no apparent difference in the body weight gain, fat mass, hepatic lipid droplets, and adipocyte size in different adipose depots between the OE and control mice (Figure S6G–J). When fed a HFD, OE mice showed less body weight gain than control mice (Figure 6H). Moreover, OE mice showed better glucose tolerance and insulin sensitivity than control mice (Figure 6I and J). OE mice showed reduced fat mass, liver mass, hepatic lipid droplets, and adipocyte size under HFD feeding (Figure 6K–N). Consistently, the serum levels of TG, TC, LDL, FFA, and BCAA decreased in OE mice (Figure S6K–L). These data indicated that BAT-specific gain of *Ybx3* could prompt thermogenesis to protect against diet-induced metabolic dysregulation.

**Figure 6:**
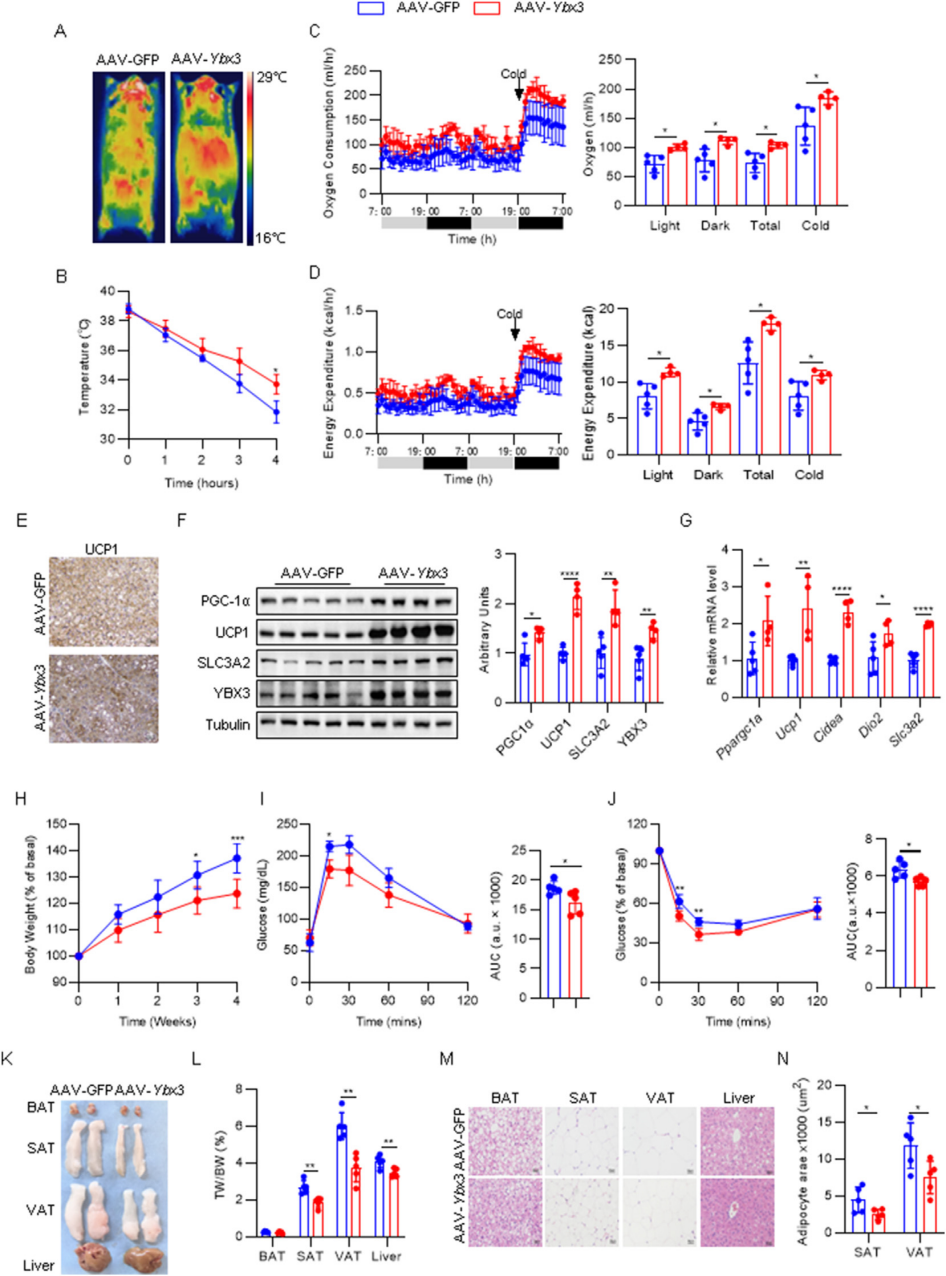
**BAT-specific gain of *Ybx3* prompts thermogenesis to protect against diet-induced metabolic dysregulation**. (A) Thermal imagery under acute cold exposure (*n* = 4–5). (B) Core temperature under cold exposure (*n* = 4–5). (C) Hourly oxygen consumption curve (left) and quantification (right) (*n* = 4–5). (D) Hourly energy expenditure curve (left) and quantification (right) (*n* = 4–5). (E) IHC staining of UCP1 in BAT after acute cold exposure (*n* = 4–5, Bar = 50 μm). (F) Immunoblots of PGC-1α, UCP1, SLC3A2, and YBX3 in the BAT after acute cold exposure (left) and quantification (right) (*n* = 4–5). (G) mRNA level of *Slc3a2* and thermogenic genes in the BAT after acute cold exposure (*n* = 4–5). (H) Body weight gain curve under HFD feeding (*n* = 5). (I–J) GTT (I) and ITT (J) under HFD feeding (*n* = 5). (K) Gross picture of BAT, SAT, VAT, and liver under HFD feeding. (L) The ratio of adipose tissue and liver weight to body weight under HFD feeding (*n* = 5). (M) H&E staining of BAT, SAT, VAT, and liver under HFD feeding (*n* = 5, Bar = 50 μm). (N) The adipocyte area quantification of SAT and VAT under HFD feeding (*n* = 5). Data are shown as mean ± SD. Hourly oxygen consumption and energy expenditure in Figures 7C and D are shown as the mean ± SEM. ∗*P* < 0.05, ∗∗*P* < 0.01, ∗∗∗*P* < 0.001, ∗∗∗∗*P* < 0.0001 by two-way ANOVA with Bonferroni multiple comparison test (B, H-J), ANCOVA with body weight as covariant (C, D) or two-tailed Student’s t-test (F-G, I-J, L, M).

## 4 Discussion

YBX family proteins are evolutionarily conserved CSD-containing proteins. The CSD was initially identified in bacterial cold-shock proteins (CSPs), which were robustly induced in response to acute low-temperature stimulation [8,9]. Previous studies have reported the temperature sensitivity and thermogenic function of YBX1 and YBX2 within SAT and BAT [4,8,10,12,30]. In this study, we characterized YBX3 as a brown adipocyte-enriched RBP responding to ambient temperature, adipogenic, and β3-adrenergic/cAMP signaling. Using AAV-mediated BAT-specific knockdown and overexpression of *Ybx3*, we demonstrated that YBX3, like other YBX members, is essential for BAT thermogenesis. However, whether all three YBX members orchestrate or counteract with each other during the cold-induced BAT thermogenesis and the potential role of YBX3 in white adipose browning were not disclosed in this study. Thus, conditional double or even triple knockout mice for YBX proteins are warranted to clarify these issues in the future.

In addition to the well-established thermogenesis function, BAT is an important metabolic sink for metabolic substrates such as glucose and fatty acids and regulates the clearance of circulation metabolites [2].In the past decade, mounting evidence has suggested that BAT is another primary metabolic sink for BCAAs. The oxidation flux of BCAAs was found to be the highest in BAT, and the total amount of oxidation in BAT was second only to skeletal muscle [26,31]. Notably, it is reported that mitochondrial SLC25A44-mediated BCAA catabolism is responsible for BAT thermogenesis [26,32]. BAT-specific deficiency of BCAA catabolic enzyme reduces circulation BCAA clearance, inhibits thermogenesis, and exacerbates diet-induced obesity and insulin resistance [26]. Therefore, targeting BAT BCAA catabolism emerges as a therapeutic opportunity for obesity and related metabolic dysfunction. However, the regulation of BCAA influx in BAT during cold exposure has not been fully elucidated. Previous studies reported that YBX3 controls cellular amino acid homeostasis by stabilizing mRNAs encoding solute carrier transporters, including BCAA transporter SLC3A2/SLC7A5 [21,24]. In this study, we unveiled that YBX3 facilitates BCAA influx of BAT and fuels brown adipocyte differentiation and cold-induced thermogenesis by stabilizing *Slc3a2* mRNA, whose encoded protein SLC3A2 forms the subunit of BCAA transporter. Besides, our results suggested a PPARγ-dependent regulation of YBX3 in BCAA catabolism and thermogenesis. These results together highlight the role of YBX3 in linking BCAA metabolism and energy control within BAT. Due to the multilevel regulation and broad binding of YBX proteins, we could not exclude the potential post-transcriptional targets or transcriptional regulatory effects of YBX3, which contribute to brown adipocyte differentiation and thermogenesis.

In summary, we illustrate the importance of cold-induced YBX3 in brown adipocyte differentiation and thermogenesis. Our results indicate PPARγ and BCAA transporter SLC3A2 as the direct targets of YBX3 orchestrating BCAA metabolism and thermogenesis, thereby highlighting a previously unknown YBX3-PPARγ-SLC3A2-BCAA axis in the regulation of BAT thermogenesis and energy balance.

## CRediT authorship contribution statement

**Lin-Yun Chen:** Writing – review & editing, Writing – original draft, Methodology, Investigation, Formal analysis. **Li-Wen Wang:** Methodology, Investigation. **Jie Wen:** Methodology, Investigation. **Jing-Dong Cao:** Methodology, Investigation. **Rui Zhou:** Methodology, Investigation. **Jin-Lin Yang:** Methodology, Investigation. **Ye Xiao:** Resources, Formal analysis. **Tian Su:** Resources, Formal analysis. **Yan Huang:** Resources, Formal analysis. **Qi Guo:** Resources, Formal analysis. **Hai-Yan Zhou:** Writing – review & editing, Funding acquisition. **Xiang-Hang Luo:** Writing – review & editing, Funding acquisition. **Xu Feng:** Writing – review & editing, Writing – original draft, Supervision, Funding acquisition, Conceptualization.

## Declaration of competing interest

The authors declare that they have no known competing financial interests or personal relationships that could have appeared to influence the work reported in this paper.

## Figures and Tables

**figs1:**
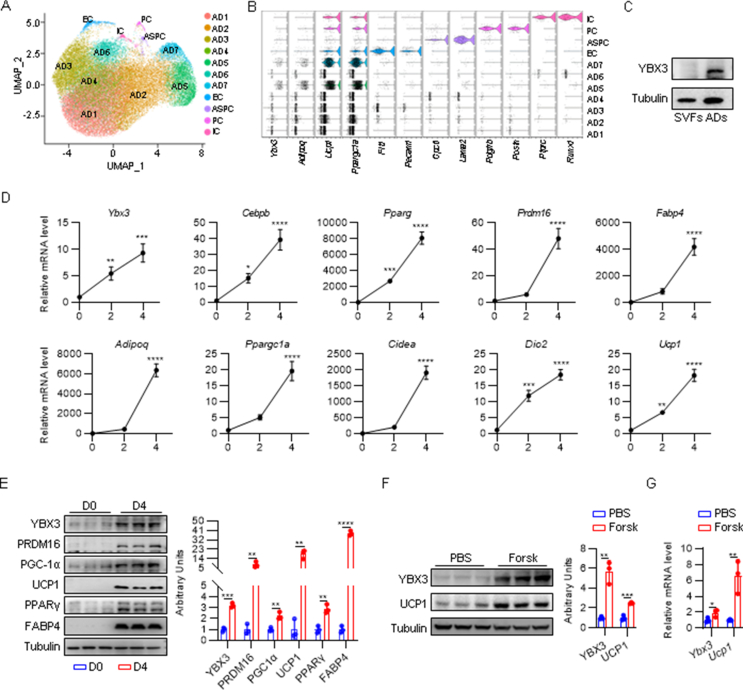


**figs2:**
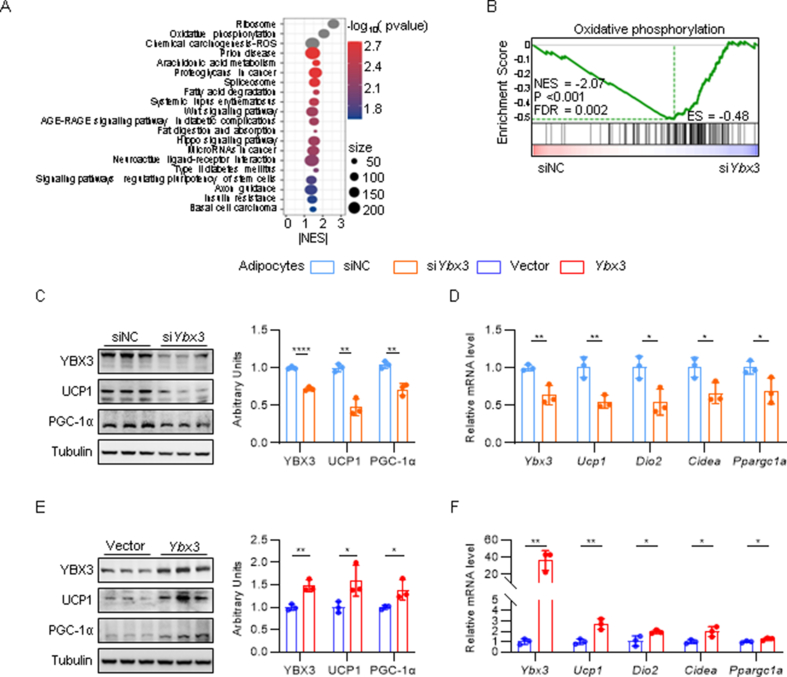


**figs3:**
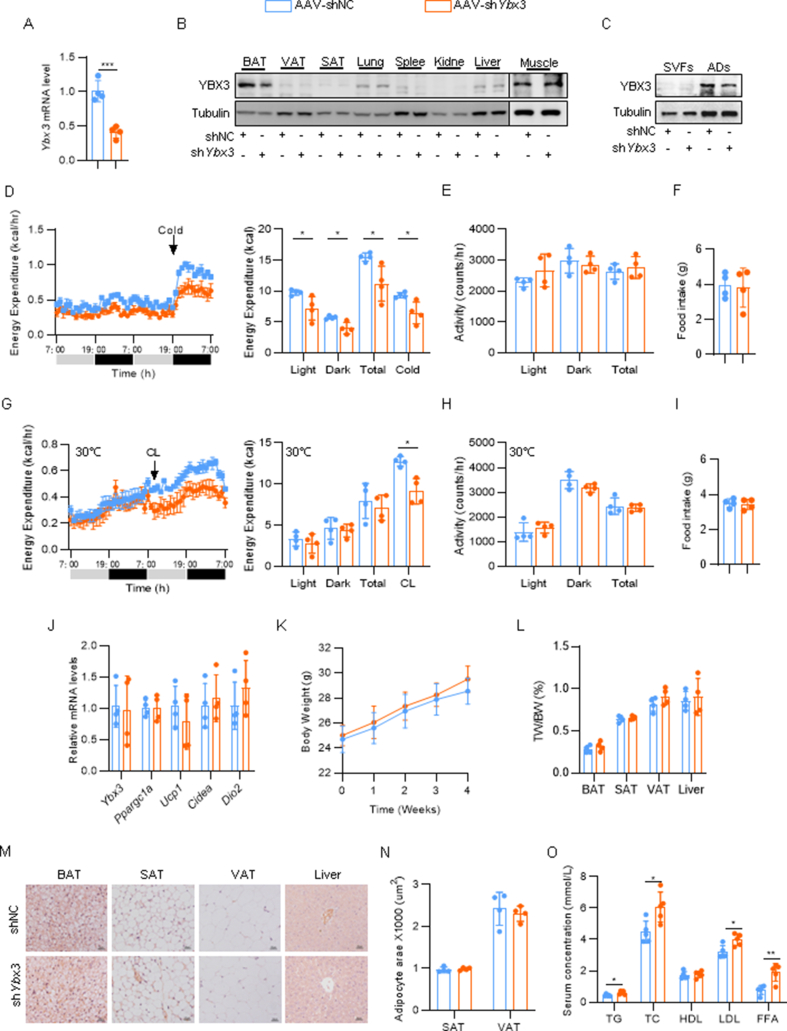


**figs4:**
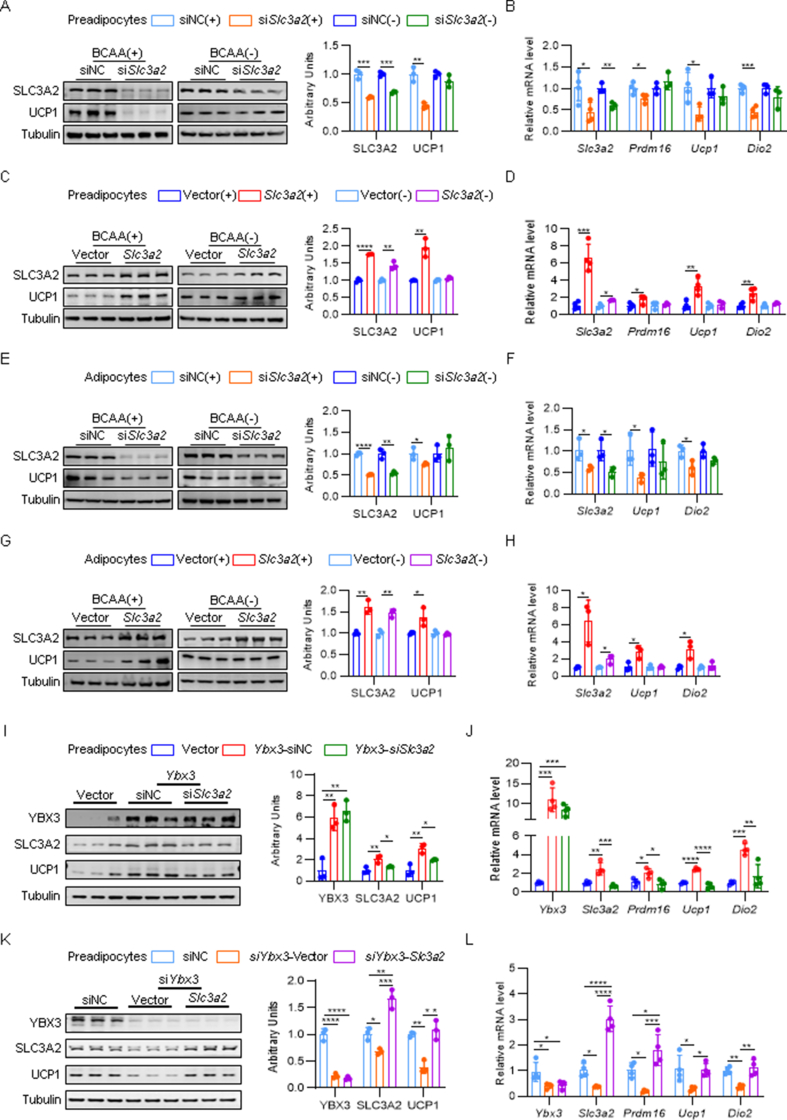


**figs5:**
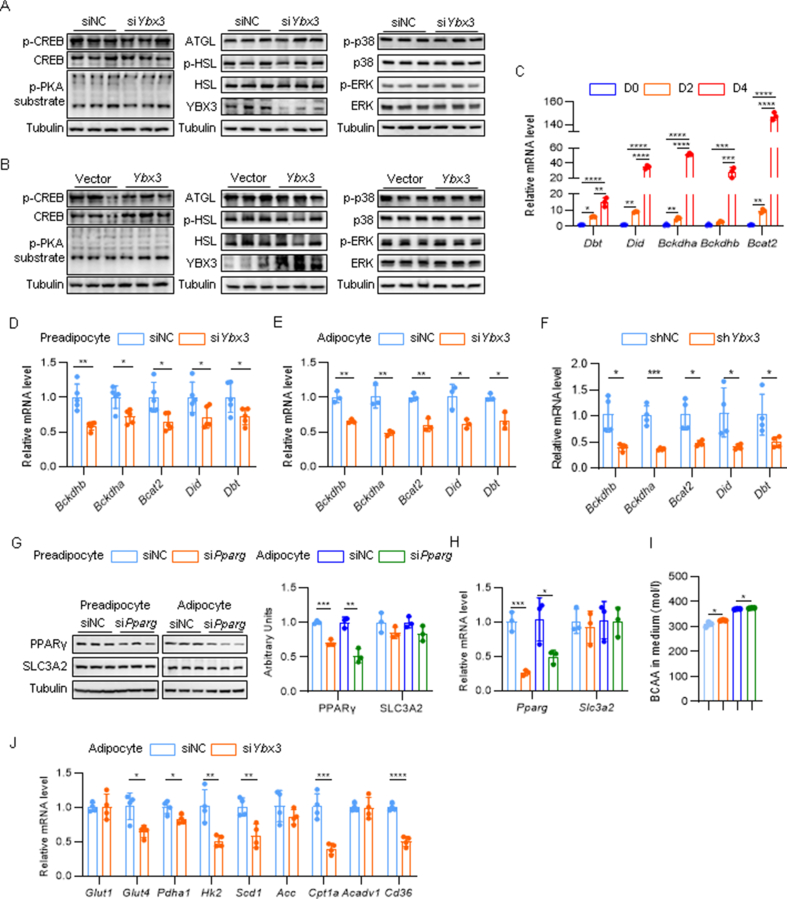


**figs6:**
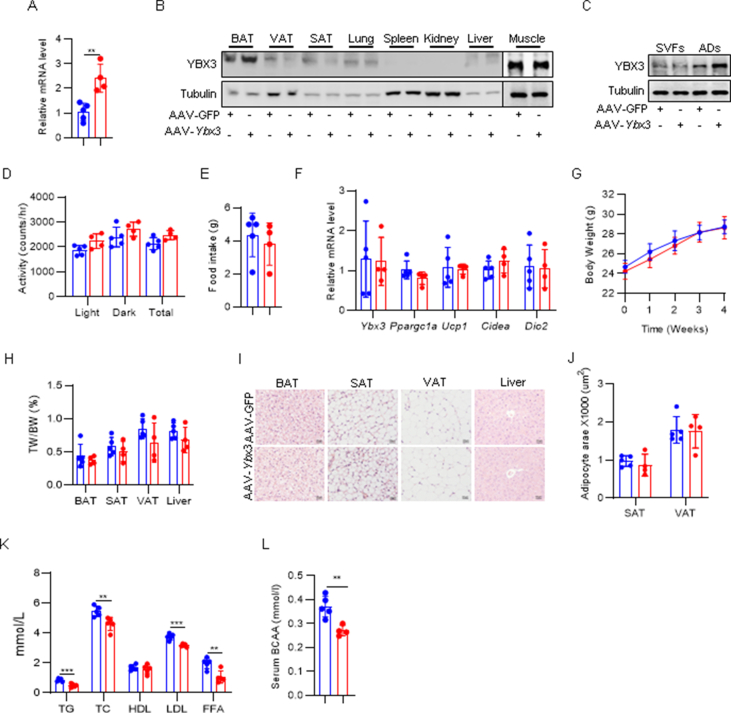


## Data Availability

The accession number for RNA-Seq data produced in this study is GSE277858. The accession numbers for the public datasets used in this study are GSE181123, GSE222424, GSE29897, and GSM7809269. The data for RBP expression profiles are acquired from BioGPS (http://biogps.org/). All other data supporting the findings of this study are available in the paper and its Supplementary Information.
