# Supplementary material for: RNA-binding protein YBX3 promotes PPARγ-SLC3A2 mediated BCAA metabolism fueling brown adipogenesis and thermogenesis
Source: Mol Metab. 2024 Oct 29;90:102053. doi: 10.1016/j.molmet.2024.102053 (PMC11570976; doi:10.1016/j.molmet.2024.102053)
Supplement: Multimedia component 1 [file mmc1.docx]

**Supplementary Material for**

**RNA-binding protein YBX3 promotes PPARγ-SLC3A2 mediated BCAA metabolism fueling brown adipogenesis and thermogenesis**

Lin-Yun Chen^1^, Li-Wen Wang^1^, Jie Wen^1,2^, Jing-Dong Cao^1^, Rui Zhou^1^, Jin-Lin Yang^1^, Ye Xiao^1,2^, Tian Su^1,2^, Yan Huang^1,2^, Qi Guo^1,2^, Hai-Yan Zhou^1,2^, Xiang-Hang Luo^1,2^, Xu Feng^1,2^

1 Department of Endocrinology, Endocrinology Research Center, Xiangya Hospital of Central South University, Changsha, Hunan, 410008, China.

2 National Clinical Research Center for Geriatric Disorders, Xiangya Hospital, Changsha, Hunan, 410008, China

* Address all correspondence and request for reprints to:

Dr. Xu Feng,

Department of Endocrinology, Endocrinology Research Center

Xiangya Hospital of Central South University

87# Xiangya Road, Changsha, Hunan 410008, PR China

E-mail: [fengxu@csu.edu.cn](mailto:fengxu@csu.edu.cn)

**Supplementary Table 1 Nucleotide sequences of primers used for qPCR analysis.**

| *β-Actin* | F: GATCATTGCTCCTCCTGAGC |
| --- | --- |
|  | R: ACTCCTGCTTGCTGATCCAC |
| *Ybx3* | F: AGAAAACCAGCAAGCGGCTA |
|  | R: TGAAACAGCGTTGAGGGGAC |
| *Ucp1* | F: AGGCTTCCAGTACCATTAGGT |
|  | R: CTGAGTGAGGCAAAGCTGATTT |
| *Cidea* | F: TGACATTCATGGGATTGCAGAC |
|  | R: GGCCAGTTGTGATGACTAAGAC |
| *Dio2* | F: AATTATGCCTCGGAGAAGACCG |
|  | R:GGCAGTTGCCTAGTGAAAGGT |
| *Ppargc1a* | F: TATGGAGTGACATAGAGTGTGCT |
|  | R: CCACTTCAATCCACCCAGAAAG |
| *Prdm16* | F: CCACCAGCGAGGACTTCAC |
|  | R: GGAGGACTCTCGTAGCTCGAA |
| *Cebpb* | F: CCGGATCAAACGTGGCTGA |
|  | R: GATTACTCAGGGCCCGGCTG |
| *Pparg* | F: TCGCTGATGCACTGCCTATG |
|  | R: GAGAGGTCCACAGAGCTGATT |
| *Adipoq* | F: TGTTCCTCTTAATCCTGCCCA |
|  | R: CCAACCTGCACAAGTTCCCTT |
| *Fabp4* | F: AAGGTGAAGAGCATCATAACCCT |
|  | R: TCACGCCTTTCATAACACATTCC |
| *Cox4i* | F: GCCTGATTGGCAAGAGAGCC |
|  | R: CAAGGGGTAGTCACGCCGAT |
| *Cox8b* | F: GCGAAGTTCACAGTGGTTCC |
|  | R: CGACTATGGCTGAGATCCCC |
| *Cox7a* | F: GCTCTGGTCCGGTCTTTTAGC |
|  | R: GTACTGGGAGGTCATTGTCGG |
| *Tfam* | F: ATTCCGAAGTGTTTTTCCAGCA |
|  | R: TCTGAAAGTTTTGCATCTGGGT |
| *Nrf1* | F: AGCACGGAGTGACCCAAAC |
|  | R: TGTACGTGGCTACATGGACCT |
| *Mfn1* | F: CCTACTGCTCCTTCTAACCCA |
|  | R: AGGGACGCCAATCCTGTGA |
| *Fis1* | F: TGTCCAAGAGCACGCAATTTG |
|  | R: CCTCGCACATACTTTAGAGCCTT |
| *Opa1* | F: TGGAAAATGGTTCGAGAGTCAG |
|  | R: CATTCCGTCTCTAGGTTAAAGCG |
| *Slc3a2* | F: TGTACTTGGCTGAGTGGCAG |
|  | R: AGGTCGCTGGTGGATTCAAG |
| *Slc7a5* | F: GCTGACGAACCTGGCCTATT |
|  | R: ACCCATTGACAGAGCCGAAG |
| *Bckdha* | F: AGGAGGTGCTGAAGTTCTACC |
|  | R: CGCCATAGTTGGTCATGTAGAAG |
| *Bckdhb* | F: AGTGCCCTGGATAACTCATTAGC |
|  | R: GCATCGGAAGACTCCACCAAA |
| *Bcat2* | F: TTCCAGAACCTCACGCTACAC |
|  | R: TAGCAGAACGTAGCATCCTGTC |
| *Did* | F: GAGCTGGAGTCGTGTGTACC |
|  | R: GAACCTATCACTGTCACGTCAG |
| *Dbt* | F: AGACTGACCTGTGTTCGCTAT |
|  | R: GAGTGACGTGGCTGACTGTA |
| ND1 | F: CTAGCAGAAACAAACCGGGC |
|  | R: CCGGCTGCGTATTCTACGTT |
| 16S | F: CCGCAAGGGAAAGATGAAAGAC |
|  | R: TCGTTTGGTTTCGGGGTTTC |
| Hk2 | F: GCCAGCCTCTCCTGATTTTAGTGT |
|  | R: GGGAACACAAAAGACCTCTTCTGG |
| *Glut1* | F: GCAGTTCGGCTATAACACTGG |
|  | R: GCGGTGGTTCCATGTTTGATTG |
| *Glut4* | F: GGACCGGATTCCATCCCAC |
|  | R: TCCCAACCATTGAGAAATGATGC |
| *Phda1* | F: GAAATGTGACCTTCATCGGCT |
|  | R: TGATCCGCCTTTAGCTCCATC |
| *Hk2* | F: GTGTGCTCCGAGTAAGGGTG |
|  | R: CAGGCATTCGGCAATGTGG |
| *Scd1* | F: TTCTTGCGATACACTCTGGTGC |
|  | R: CGGGATTGAATGTTCTTGTCGT |
| *Acaca* | F: ATGGGCGGAATGGTCTCTTTC |
|  | R: TGGGGACCTTGTCTTCATCAT |
| *Cpt1a* | F: CTCCGCCTGAGCCATGAAG |
|  | R: CACCAGTGATGATGCCATTCT |
| *Acadvl* | F: TGACCTTGGTGTTAGCGTTAC |
|  | R: CTGGGCCTTTGTGCCATAGAG |
| Cd36 | F: AGATGACGTGGCAAAGAACAG |
|  | R: CCTTGGCTAGATAACGAACTCTG |

**FigureS1. YBX3 is a brown adipocyte-enriched RBP responding to ambient temperature and adrenergic signaling.**

(A) UMAP plot of single-nucleus RNA-seq data of BAT. IC, immune cells; PC, pericyte; ASPC, adipose stem and progenitor cells; EC, endothelial cells; AD, adipocytes.

(B) Violin plot shows key marker gene expression of different cell clusters in (A).

(C) Immunoblot of YBX3 in the adipocytes and SVF cells isolated from BAT (n = 3).

(D) mRNA levels of *Ybx3*, adipogenic and thermogenic genes during brown adipocyte differentiation at indicated time (n = 3).

(E) Immunoblot of YBX3, adipogenic, and thermogenic proteins in preadipocytes and differentiated brown adipocytes (left) and quantification (right) (n = 3).

(F) Immunoblot of YBX3 and UCP1 in the brown adipocytes treated with vehicle or forskolin (10uM) for 24h (left) and quantification (right) (n = 3).

(G) mRNA levels of *Ybx3* and *Ucp1* in the brown adipocytes treated with vehicle or forskolin (10μM) for 24h (n = 3).

Data are shown as the mean ±SD. *p < 0.05, **P < 0.01, ***P < 0.001, ****P < 0.0001 by one-way ANOVA with Dunnett multiple comparisons test (D) or two-tailed Student's t-test (C, E-G).

**FigureS2. YBX3 is essential for brown adipocyte differentiation and thermogenesis in vitro.**

(A) Bubble Chart shows the most affected KEGG pathway in *Ybx3* knockdown brown adipocytes versus control cells.

(B) GSEA enrichment plot of oxidative phosphorylation pathway in *Ybx3* knockdown brown adipocytes and control cells.

(C, E) Immunoblots of YBX3 and thermogenic proteins in mature brown adipocytes with *Ybx3* knockdown(C) or overexpression (E) brown adipocytes and control cells (left), and quantification (right) (n = 3).

(D, F) mRNA levels of *Ybx3* and thermogenic genes in mature brown adipocytes with *Ybx3* knockdown (D) or overexpression (F) and control cells (n = 3).

Data are shown as the mean ±SD. *p < 0.05, **P < 0.01, ****P < 0.0001 by two-tailed Student's t-test (C-F).

**FigureS3. BAT-specific loss of *Ybx3* impairs thermogenesis and exacerbates diet-induced obesity.**

(A) mRNA levels of *Ybx3* in the BAT under ND feeding (n = 4).

(B) Immunoblot of YBX3 in indicated tissues (n = 3).

(C) Immunoblot of YBX3 in the adipocytes and SVF cells isolated from BAT (n = 3).

(D) Hourly energy expenditure curve (left) and quantification (right) under ND feeding (n = 4).

(E-F) Physical activity (E) and food intake (F) under ND feeding (n = 4).

(G) Hourly energy expenditure curve (left) and quantification (right) after CL treatment (n=4).

(H-I) Physical activity (H) and food intake (I) after CL treatment (n=4).

(J) mRNA levels of thermogenic genes in the SAT after acute cold exposure (n = 4).

(K) Body weight gain curve under ND feeding (n = 9).

(L) The ratio of adipose tissue and liver weight to body weight under ND feeding (n = 4).

(M) H&E staining of BAT, SAT, VAT, and liver under ND feeding (n = 4, Bar = 50μm).

(N) The adipocyte area quantification of SAT and VAT under ND feeding (n = 4).

(O) Serum levels of TG, TC, HDL, LDL, and FFA under HFD feeding (n = 5)

Data are shown as the mean ± SD or mean ± SEM (D and G). *p < 0.05, **P < 0.01,***P < 0.001 by two-tailed Student's t-test (A, O), ANCOVA with body weight as covariant(D, G)

**FigureS4.YBX3 stabilizes *Slc3a2* mRNA to facilitate BCAA influx and fuel brown adipocyte differentiation and thermogenesis.**

(A) Immunoblots of SLC3A2 and UCP1 in *Slc3a2* knockdown brown adipocytes and control cells with or without BCAA (left) and quantification (right) (n = 3).

(B) mRNA level of *Slc3a2* and thermogenic genes in *Slc3a2* knockdown brown adipocytes and control cells with or without BCAA (n = 3-4).

(C) Immunoblots of SLC3A2 and UCP1 in *Slc3a2* overexpressed brown adipocytes and control cells with or without BCAA (left) and quantification (right) (n = 3).

(D) mRNA level of *Slc3a2* and thermogenic genes in *Slc3a2* overexpressed brown adipocytes and control cells with or without BCAA (n = 3-4).

(E) Immunoblots of SLC3A2 and UCP1 in mature brown adipocytes with *Slc3a2* knockdown and control cells with BCAA or without BCAA (left) and quantification (right) (n = 3).

(F) mRNA level of *Slc3a2* and thermogenic genes in mature brown adipocytes with *Slc3a2* knockdown and control cells with or without BCAA (n = 3).

(G) Immunoblots of SLC3A2 and UCP1 in mature brown adipocytes with *Slc3a2* overexpression and control cells with or without BCAA (left) and quantification (right) (n = 3).

(H) mRNA level of *Slc3a2* and thermogenic genes in mature brown adipocytes with *Slc3a2* overexpression and control cells with or without BCAA (n = 3).

(I) Immunoblots of YBX3, SLC3A2, and UCP1 in brown adipocytes with indicated treatment(left) and quantification (right) (n = 3).

(J) mRNA level of *Ybx3*, *Slc3a2* and thermogenic genes in brown adipocytes with indicated treatment (n = 4).

(K) Immunoblots of YBX3, SLC3A2, and UCP1 in brown adipocytes with indicated treatment(left) and quantification (right) (n = 3).

(L) mRNA level of *Ybx3*, *Slc3a2* and thermogenic genes in brown adipocytes with indicated treatment (n = 4).

Data are shown as the mean ±SD. *p < 0.05, **P < 0.01, ***P < 0.001, ****P < 0.0001 by two-tailed Student's t-test (A-H), one-way ANOVA with Tukey multiple comparison test (I-L).

**FigureS5. Loss of *Ybx3* disturbs PPARγ-dependent brown adipocyte thermogenesis and BCAA catabolism**

(A-B)Immunoblots of p-CREB, CREB, p-PKA substrates, ATGL, p-HSL, HLS, p-p38, p38, p-ERK and ERK in mature brown adipocytes with *Ybx3* knockdown (A) or overexpression (B) and control cells (n = 3).

(C) mRNA level of genes related to BCAA transport and catabolism during brown adipocyte differentiation (n = 3).

(D) mRNA level of BCAA catabolism genes in *Ybx3* knockdown brown adipocytes and control cells (n = 5).

(E) mRNA level of BCAA catabolism genes in mature brown adipocytes with *Ybx3* knockdown and control cells (n = 3).

(F) mRNA level of BCAA catabolism genes in BAT under ND feeding (n= 4).

(G) Immunoblots of PPARγ and SLC3A2 in *Pparg* knockdown brown adipocytes and mature brown adipocytes with *Pparg* knockdown (left) and quantification (right) (n = 3).

(H) mRNA level of *Pparg* and *Slc3a2* in *Pparg* knockdown brown adipocytes and mature brown adipocytes with *Pparg* knockdown (n = 3).

(I) BCAA levels in medium from *Pparg* knockdown brown adipocytes and mature brown adipocytes with *Pparg* knockdown (n = 3).

(J) mRNA levels of glucose and fatty acid metabolic genes in mature brown adipocytes with *Ybx3* knockdown and control cells (n = 4).

Data are shown as the mean ±SD. *p < 0.05, **P < 0.01, ***P < 0.001, ****P < 0.0001 by two-tailed Student's t-test (D-J) or one-way ANOVA with Tukey multiple comparison test (C).

**Figure S6 BAT-specific gain of *Ybx3* prompts thermogenesis to protect against diet-induced metabolic dysregulation.**

(A) mRNA level of *Ybx3* in the BAT (n = 4-5).

(B) Immunoblot of YBX3 in indicated tissues (n = 3).

(C) Immunoblot of YBX3 in the adipocytes and SVF cells isolated from BAT (n = 3).

(D-E) Physical activity(D) and food intake(E) under ND feeding(n = 4-5).

(F) mRNA level of *Ybx3* and thermogenic genes in the SAT after acute cold exposure (n = 4-5).

(G) Body weight of mice injected with AAV-GFP or AAV-*Ybx3* under ND feeding (n = 4-5).

(H) The ratio of adipose tissue weight and liver to body weight under ND feeding (n = 4-5).

(I) H&E staining of BAT, SAT, VAT, and live under ND feeding (n = 4-5, Bar = 50μm).

(J) The adipocyte area quantification of SAT and VAT under ND feeding (n = 4-5).

(K) Serum levels of TG, TC, HDL, LDL, and FFA under HFD feeding (n = 5).

(L) Serum BCAA levels under HFD feeding (n= 4-5).

Data are shown as the mean ±SD. **P < 0.01, ***P < 0.001 by two-tailed Student's t-test (A, K-L).
